# Therapeutic Potential of Tacrolimus on Acute Myocardial Infarction in Minipigs: Analysis with Serial Cardiac Magnetic Resonance and Changes at Histological and Protein Levels

**DOI:** 10.1155/2014/524078

**Published:** 2014-07-09

**Authors:** Sheung-Fat Ko, Hon-Kan Yip, Steve Leu, Chen-Chang Lee, Jiunn-Jye Sheu, Chia-Chang Lee, Shu-Hang Ng, Chung-Cheng Huang, Min-Chi Chen, Cheuk-Kwan Sun

**Affiliations:** ^1^Department of Radiology, Kaohsiung Chang Gung Memorial Hospital, Chang Gung University, College of Medicine, 123 Ta-Pei Road, Niao-Sung District, Kaohsiung 833, Taiwan; ^2^Department of Cardiology, Kaohsiung Chang Gung Memorial Hospital, Chang Gung University, College of Medicine, 123 Ta-Pei Road, Niao-Sung District, Kaohsiung 833, Taiwan; ^3^Center for Translational Research in Biomedical Sciences, Kaohsiung Chang Gung Memorial Hospital, Chang Gung University, College of Medicine, 123 Ta-Pei Road, Niao-Sung District, Kaohsiung 833, Taiwan; ^4^Department of Cardiovascular Surgery, Kaohsiung Chang Gung Memorial Hospital, Chang Gung University, College of Medicine, 123 Ta-Pei Road, Niao-Sung District, Kaohsiung 833, Taiwan; ^5^Public Health and Biostatistics Center, Kaohsiung Chang Gung Memorial Hospital, Chang Gung University, College of Medicine, 123 Ta-Pei Road, Niao-Sung District, Kaohsiung 833, Taiwan; ^6^Department of Emergency Medicine, E-Da Hospital, I-Shou University, Number 1, Yi-Da Road, Kaohsiung 82445, Taiwan

## Abstract

This study investigates the therapeutic potential of intracoronary tacrolimus against acute myocardial infarction (AMI) in minipigs with serial cardiac magnetic resonance (CMR) and changes at histological and protein levels. Twelve minipigs subjected to permanent left anterior descending artery ligation were randomized as tac-treated group (*n* = 6, with intracoronary tacrolimus treatment) and controls (*n* = 6). CMR with cine and late gadolinium enhancement (LGE) studies were performed on postoperative days 2, 5, and 21. There were no significant differences in left ventricular function (LVF), contractility, and LGE between the two groups on day 2. On day 5, the tac-treated group showed a significantly higher ejection fraction, smaller infarct, and lower day-5/day-2 infarct ratio than controls. On day 21, the controls demonstrated further deterioration of LVF and infarct. Contrastingly, the tac-treated animals demonstrated preservation of LVF, contractility, significantly smaller infarct, and lower day-21/day-2 infarct ratios compared with those on day 5 and controls. The *in vivo* CMR results were correlated with *in vitro* findings on histology, immunostaining, and Western blotting which revealed significantly less fibrosis, higher vascularities, less CD68+ and CD40+ inflammatory cells, lower expressions of inflammatory (MMP-9, NF-*κ*B, and TNF-*α*), and apoptotic (Bax, Caspase-3, c-PARP) biomarkers, respectively, in tac-treated AMI minipigs than controls.

## 1. Introduction

Ischemic heart disease is one of the leading causes of death worldwide [1]. Early reperfusion of the jeopardized myocardium remains the most common approach to treating acute myocardial infarction (AMI) [2]. However, reperfusion harbors potential shortcomings, including myocardial stunning, ventricular arrhythmia, and microvascular obstruction [3]. Recent studies have proposed that inflammatory and immune reactions also play important roles in AMI and that administrating immunosuppressive drugs to block such reactions could potentially salvage the myocardium [4, 5]. Experimental and clinical studies have reported that cyclosporine, a potent immunosuppressant, can reduce left ventricular (LV) infarct size and preserve LV function (LVF) after AMI [6, 7]. Tacrolimus has been reported to be more potent than cyclosporine in immunosuppression and positive vascular remodeling [8, 9]. Two recent studies reported preserved LVF in minipigs with AMI treated by intracoronary administration of tacrolimus using echocardiographic assessment [9, 10]. However, the infarct* per se*, a major determinant of mortality in AMI, cannot be easily detected by echocardiography [7]. In contrast, a late gadolinium enhancement (LGE) study on cardiac magnetic resonance (CMR) is accurate for the assessment of infarct size [11–14]. The present study investigates the therapeutic potential of intracoronary tacrolimus administration in minipigs after permanent left anterior descending (LAD) artery ligation using serial CMR with detailed* in vivo* assessment of left ventricular changes. The findings from* in vitro* histopathologic, immunostaining, and Western blot studies of the explanted heart were used for confirming the* in vivo *observations.

## 2. Material and Methods

### 2.1. Ethics Statement

This study was carried out in strict accordance with the recommendations in the Guide for the Care and Use of Laboratory Animals of the National Institutes of Health. The protocol was approved by* Institutional Committee on Animal Care, Use and Research* (Approval no. 2011070502) of the Kaohsiung Chang Gung Memorial Hospital and Chang Gung University College of Medicine. All surgery was performed under ketamine and isoflurane anesthesia, and all efforts were made to minimize suffering.

### 2.2. Surgical Procedure

After anesthesia using ketamine (15 mg/kg, intramuscular injection) and inhalation of 1.5% isoflurane, the male minipig (Livestock Research Institute, Taitung, Taiwan), weighing 16–19 kg, was placed in supine position on a warming pad. After intubation, intravenous amiodarone (150 mg) was administered for the prevention of malignant arrhythmia. After a midthoracotomy and gentle heart exposure, the LAD was dissected free just distal to the first diagonal branch and was repeatedly ligated with 5-0 prolene sutures. AMI was confirmed by the S-T segment elevation on electrocardiogram and cyanosis of the anterior LV myocardium. After mid-LAD ligation, 6 minipigs (tac-treated group) received intracoronary injections of tacrolimus (0.5 mg in 2.5 mL physiological saline) through the LAD with a 22-guage needle inserted just beyond the point of ligation. After infusion of tacrolimus (which is oily), slow saline flushing was applied and adequate tacrolimus diffusion could be confirmed by observation of lightening of the discoloration of cyanotic myocardium and occurrence of mildly oily or shiny appearance of LV surface. In the other 6 minipigs (the control group), an equal amount of physiological saline was injected. The muscle and skin of the chest wall were closed. The animals were allowed to recover on the warming pad.

### 2.3. Rationale of Drug Dosage

The tacrolimus dosage (0.5 mg/kg) was based on a preliminary trial in 6 minipigs in which tacrolimus was administered at three different dosages (1.0 mg/kg; 0.5 mg/kg; 0.25 mg/kg; each in two minipigs). Two minipigs receiving the highest dosage (1.0 mg/kg) had fatal malignant ventricular tachyarrhythmia. Although no complications were noted in the minipigs with the two other dosages, the infarct size was remarkably smaller in animals receiving 0.5 mg/kg as revealed in the explanted heart after the animals were euthanized. Therefore, this dosage was utilized in the current study.

### 2.4. Cardiac Magnetic Resonance

#### 2.4.1. CMR Protocols

All 12 minipigs underwent CMR on days 2, 5, and 21 after LAD ligation performed by 1 investigator using a 1.5 T MR imager (Discovery MR450; GE Healthcare, Milwaukee, WI). Of note, the growth rate of minipig is about 5-6 times of human being and thus a 21-day follow-up in minipig is approximately equal to a 15- to 18-week duration in human. After anesthesia, the minipig was placed in a supine position and a phased array coil was wrapped around the chest. Venous access was achieved via an ear vein. CMR were done in short-axis view of the LV and the parameters are summarized in Table 1. A 2-dimensional FIESTA-gated-CINE technique was used for the assessment of LV volume/mass, LVF and regional function. Afterwards, 0.2 mmol/kg body weight (BW) gadopentetate dimeglumine (Magnevist/Bayer-Schering Pharma, Berlin) was administered at 0.6 mL/s via the ear vein with a power injector (Spectris/Medrad, Indianola, PA) followed by a 5 mL saline flush. Twelve minutes after contrast injection, a 2-dimensional GRE-inversion recovery technique with gating was performed for LGE studies.

#### 2.4.2. Image Analysis

All images were evaluated by 2 experienced investigators (blinded to animal groups) by consensus with an Advantage Workstation (AW-ReportCARD 4.0, GE Healthcare). The end-systole (ES) and end-diastole (ED) volume, stroke volume (SV), and ejection fraction (EF) were measured. LV mass was calculated using the specific gravity of cardiac muscle (1.05 mg/mL) and functional parameters were normalized to BW (g/kg or mL/kg), respectively, to account for the variations in BWs at different time points. For analysis of regional wall motion or contractility, the LV was divided into 8 segments per slice with the starting point at the posterior interseptal groove and clockwise rotation on 3 short axis slices and, in order to reduce partial volume effects, the basal and apical short-axis slices were not included into analysis as suggested by previous studies [13, 14]. For each segment of the above described model, contractility was assessed on a 5-point scale (0 = normal, 1 = mild/moderate hypokinesia, 2 = severe hypokinesia, 3 = akinesia, 4 = dyskinesia) and expressed as a mean score of segments 1 to 8 of the 3 slices. For measuring infarct transmurality (percentage of infarct in 8 segments of LV wall) in controls and tac-treated animals, the same three slices used for wall motion measurements were used. The infarct area was identified as a hyperenhanced area with signal intensity >5.0 standard deviations (SD) of the normal remote myocardium in order to avoid overestimating the infarct size* in vivo* scanning [15]. The total extent of infarct on the LGE images was measured and expressed as a mean percentage of the LV area. The day 5/day 2 (D5/D2) infarct ratio (IR) and day 21/day 2 (D21/D2) IR, which were defined as the extent of LGE on day 5 and day 21 divided by the extent of LGE on day 2, respectively, were also determined.

### 2.5. Assessments of Infarct Size and Degree of Fibrosis of the Explanted Heart

Minipigs were euthanized on day 21. The explanted heart was cleaned, sliced (1 cm), stained with 2% triphenyltetrazolium chloride (TTC), and then photographed. Samples were then obtained from the infarct area, peri-infarct area, and normal remote myocardium for histologic and immunostaining studies. The total infarct volume/LV volume (%) on TTC staining and fibrotic changes on Masson trichrome staining were determined as previously described [10, 13]. The mean fibrotic area in LV myocardium per high-power-field was obtained using a conversion factor of 19.24 (1 *μ*m^2^ = 19.24 pixels).

### 2.6. Immunostaining for Vascularities and Inflammatory Cells

Immunohistochemical (IHC) staining for blood vessels, immunohistofluorescent (IHF) staining for CD68+ and IHC staining for CD40+ cells were performed as previously described [13, 14]. For each animal, 3 sections of the infarct area were chosen and 3 randomly selected high-power fields (×200) were analyzed for each section. The mean numbers of *α*-smooth muscle actin (*α*-SMA) positive small vessels (≤25 *μ*m) were determined by summing all numbers divided by 9 (for a total of 9 high-power fields in 3 slices were assessed). Percentage of CD68+ cells, an indicator of macrophages, was calculated as CD68+ cell number/4′,6-diamidino-2-phenylindole (DAPI) counter-stained nuclei number. Percentage of CD40+ cells, an indicator of inflammatory cells, was calculated as CD40+ cell number/hematoxylin counter-stained nuclei number.

### 2.7. Western Blotting

Equal amounts (50 *μ*g) of protein extracts were loaded and separated by sodium dodecyl sulfate-polyacrylamide gel electrophoresis (SDS-PAGE) using 12% acrylamide gradients. After electrophoresis, the separated proteins were transferred electrophoretically to a polyvinylidene difluoride (PVDF) membrane (Amersham Biosciences). Nonspecific sites were blocked by incubation of the membrane in blocking buffer [5% nonfat dry milk in T-TBS (TBS containing 0.05% Tween 20)] overnight. The membranes were incubated with the indicated primary antibodies [matrix metalloproteinase (MMP)-9 (1 : 5000, Abcam), nuclear factor (NF)-*κ*B (1 : 600, Abcam), tumor necrosis factor (TNF)-*α* (1 : 1000, Cell Signaling), Bax (1 : 1000, Abcam), caspase-3 (Csp-3) (1 : 4000, Abcam), cleaved poly-ADP-ribose polymerase (c-PARP) (1 : 1000, Cell Signaling), and actin (1 : 10000, Chemicon)] for 1 hr at room temperature. Horseradish peroxidase-conjugated anti-rabbit immunoglobulin IgG (1 : 2000, Cell Signaling) was used as the secondary antibody for 1 hr at room temperature. The washing procedure was repeated 8 times within 1 hr, and immunoreactive bands were visualized by enhanced chemiluminescence (ECL; Amersham Biosciences) and exposure to Biomax L film (Kodak). For purposes of quantification, ECL signals were digitized using Labwork software (UVP).

### 2.8. Statistical Analysis

Data were expressed as mean ± SD or percentage. CMR data with multiple comparisons (between-groups on 3 different days and within-group among days 2, 5, and 21) were assessed using Mann-Whitney *U* test or Wilcoxon signed-rank test (SAS for Windows, 8.2, SAS Institute, Cary, NC) with Bonferroni correction (statistically significant if *P*-value <0.017). For comparisons of D5/D2 and D21/D2 IR, a *P* value <0.05 was considered significant. The histologic and laboratory data among two groups were compared using one-way ANOVA followed by Tukey's multiple comparison procedure (statistically significant if *P*-value <0.05).

## 3. Results

### 3.1. Cardiac Magnetic Resonance

**Figure 1:**
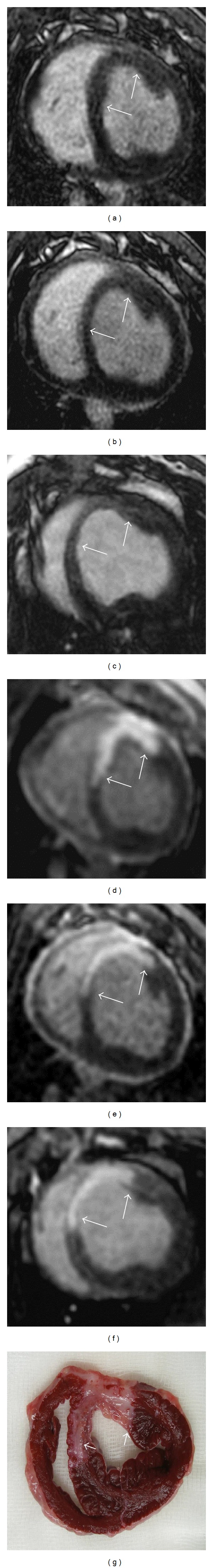
Cine (a)–(c) and LGE (d)–(f) CMR (in short-axis at midventricular level) of the minipig in the control group on postoperative days 2, 5, and 21 and TTC-stained gross specimen (g). Note the normal anteroseptal LV wall thickness (arrows) on day 2 (a), mild thinning (arrows) on day 5 (b), and further reduction in wall thickness (arrows) and dilated LV chamber on day 21 (c). LGE images demonstrate marked transmural enhancement (arrows) of anteroseptal wall on day 2 (d), persistent transmural enhancement (arrows) on day 5 (e), and further progression of transmural enhancement (arrows) on day 21 (f) with good correlation to gross specimen with extensive transmural infarction (arrows).

**Figure 2:**
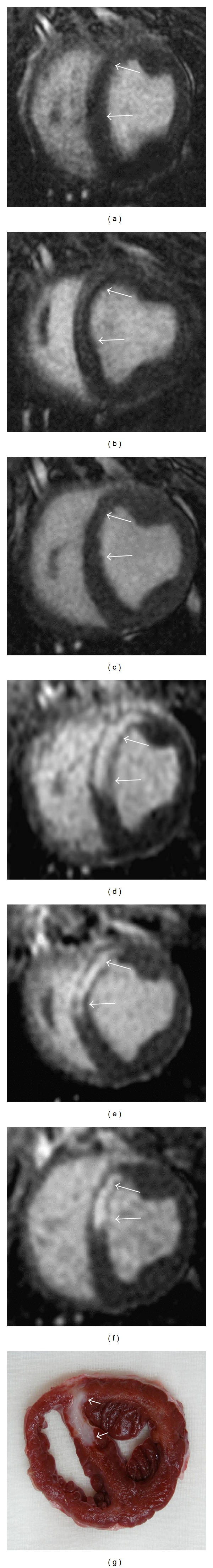
Cine (a)–(c) and LGE (d)–(f) CMR (in short-axis at midventricular level) of the minipig in the tac-treated group on postoperative days 2, 5, and 21 and TTC-stained gross specimen (g). Note the normal anteroseptal LV wall thickness (arrows) on day 2 (a), near normal LV wall (arrows) on day 5 (b), and mild reduction in LV wall thickness (arrows) and normal LV chamber on day 21 (c). LGE images demonstrate marked transmural enhancement (arrows) of anteroseptal wall on day 2 (d), reduced transmural enhancement (arrows) on day 5 (e), and further regression of transmural enhancement (arrows) on day 21 (f) with good correlation to the infarct in gross specimen (arrows).

The results of CMR are summarized in Table 2. On postoperative day 2, there were no significant differences between the control and tac-treated groups in LV mass/BW ratio, LVED, and LVES volume/BW ratios, SV and EF, and the infarct extent on LGE. Compared with day 2, the controls showed a trend of impairment of LVF with higher ED and ES volume/BW ratios, lower EF, and larger infarct on day 5 whilst there were no significant differences among the tac-treated animals. Additionally, on day 5, the control group showed significantly lower EF and larger infarct than those in the tac-treated group. From day 5 to day 21, the control group showed further loss of LV mass, deterioration of LVF, and progression of infarct (Table 2, Figure 1). During the same period, the tac-treated animals exhibited preservation of LV mass, LVF, and regression of infarct (Table 2, Figure 2). It is worth noting that tac-treated animals showed significantly higher LV mass/BW ratio, better LVF, and smaller infarct on day 21 compared to those in the controls.

**Figure 3:**
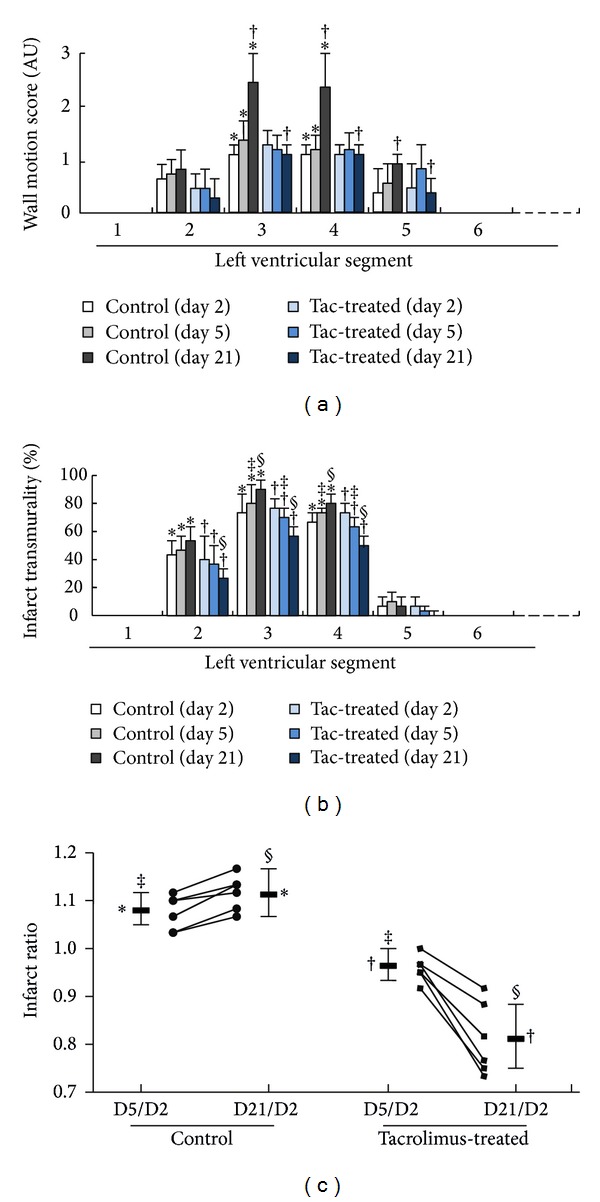
LV contractility, infarct transmurality, and infarct ratios. (a) LV contractility. Mild-to-moderate hypokinesia noted in segments 2–5 in both groups with no significant differences in intragroup comparisons between day 2 and day 5 and intergroup comparison between two groups. Note the tendency of progressive deterioration in LV contractility in segments 3 and 4 on day 21 compared to those on day 2 and day 5 in controls (^∗^ *P* = 0.031 to 0.045), whereas no significant differences in LV contractility noted in all segments between day 2, day 5, and day 21 in tac-treated minipigs. However, significantly better contractility demonstrated in segments 3–5 in the tac-treated group compared to the control group on day 21 († all *P* < 0.002). (b) Infarct transmurality. On postoperative day 2 in both groups, 40–75% transmural infarction noted in segments 2–4 without significant differences between two groups. The control group showed a tendency of progression of infarct transmurality in segments 2–4 (^∗^ *P* = 0.031 to 0.045), whereas the tac-treated group show a tendency of decline in infarct transmurality (^†^ *P* = 0.031 to 0.045) from day 2 to day 21. Compared with controls, tac-treated minipigs already showed a tendency of lower infarct transmurality in segments 3-4 on day 5 (^‡^ *P* = 0.031 to 0.045) and by day 21, significantly lower infarct transmurality was noted in segment 2–4 (§ all *P* < 0.002). (c) Infarct ratio. The control group showed significantly higher day 21/day 2 than day 5/day 2 infarct ratio (IR) (^∗^ *P* < 0.05), whereas the tac-treated group showed significant decline in infarct ratio (^†^ *P* < 0.05). Compared with the tac-treated group, the day 5/day 2 and day 21/day 2 IR were significantly higher in the control group (^‡^^§^ *P* < 0.05).

The data for LV contractility and infarct transmurality in the 8 ventricular segments on posterior operative day 2, day 5, and day 21 in the control and tac-treated groups are illustrated in Figures 3(a) and 3(b), respectively. Both groups showed impaired wall motion in segments II, III, IV, and V with no significant intragroup or intergroup differences on day 2 and day 5. On day 21, the control group showed a trend of progressive deterioration of contractility in segments III and IV and significantly poorer contractility in segments III, IV, and V compared to that of the tac-treated group (Figure 3(a)). The control group showed a trend of progressive increment in infarct transmurality in segments II, III, and IV from day 2 to day 21. Conversely, the tac-treated group showed progressive decrement in infarct transmurality in these segments (Figure 3(b)). Compared with the control group, the tac-treated group showed significantly lower infarct transmurality in segments II and III on day 5 and further regression of transmural infarct in segments II, III, and IV on day 21. All minipigs in the control group, but none in the tac-treated group, exhibited D5/D2 and D21/D2 IR > 1 in the LGE study and there were significantly higher D5/D2 and D21/D2 IR in the control group than those in the tac-treated group (Figure 3(c)).

### 3.2. Infarct Size, Degree of Fibrosis, Vascular Densities, and Inflammatory Cells in Explanted Heart

**Figure 4:**
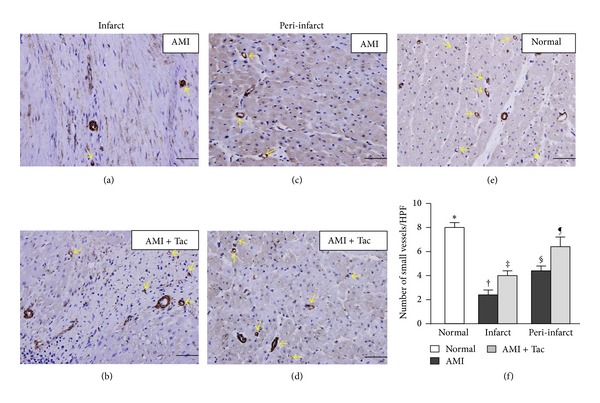
Immunohistochemical staining with *α*-smooth muscle actin for quantification of the blood vessels in myocardium in the AMI area of the control (a), AMI area of the tac-treated (b), peri-AMI area of the control (c), peri-AMI area of the tac-treated (d), and normal groups (e). Note the significantly more small vessels (≤25 *μ*m) (arrows) in normal than in the controls and the tac-treated minipigs (∗  versus other groups, all *P* < 0.05), and significantly reduced small vessels in the controls than in the tac-treated minipigs († versus ‡, § versus ¶, all *P* ≤ 0.05) (f). Scale bars in right lower corner represent 20 *μ*m.

**Figure 5:**
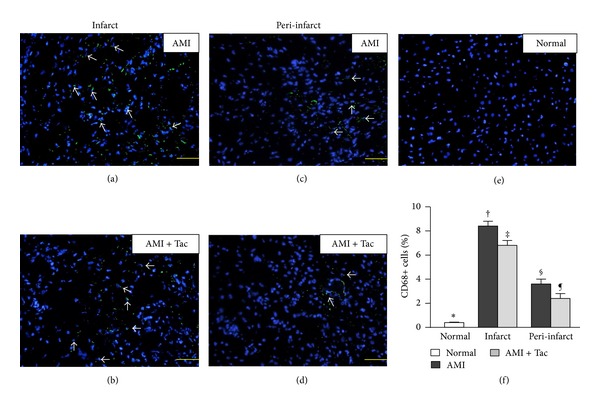
Immunohistofluorescent staining (a)–(f) for quantification of CD68+ cells in myocardium in the AMI area of the control (a), AMI area of the tac-treated (b), peri-AMI area of the control (c), peri-AMI area of the tac-treated (d), and normal groups (e). Note the significantly lower percentages of CD68+ cells (macrophages) in normal than in the controls and tac-treated minipigs (∗ versus other groups, all *P* < 0.05), and significantly higher percentages of CD68+ cells in the controls than in the tac-treated minipigs († versus ‡, § versus ¶, all *P* ≤ 0.05) (f). Scale bars in right lower corner represent 20 *μ*m.

The mean total infarct/LV volume% measured in gross specimens with TTC staining was significantly smaller in tac-treated animals than controls (11.4 ± 0.7% versus 15.7 ± 1.1%, *P* = 0.003). Masson trichrome, IHC with *α*-SMA (Figure 4), IHF for CD68+ (Figure 5), and IHC for CD40+ stainings showed significantly smaller fibrotic areas, more small vessels, lower percentages of CD68+ (macrophages), and CD40+ (inflammatory cells) cells in normal remote myocardium than in controls and tac-treated animals but as significantly greater fibrotic areas, reduced small vessels, and higher percentages of CD68+ and CD40+ cells in controls than in tac-treated minipigs, respectively, (all *P* < 0.05) (Table 3).

### 3.3. Levels of Protein Expression of Inflammatory and Apoptotic Biomarkers

**Figure 6:**
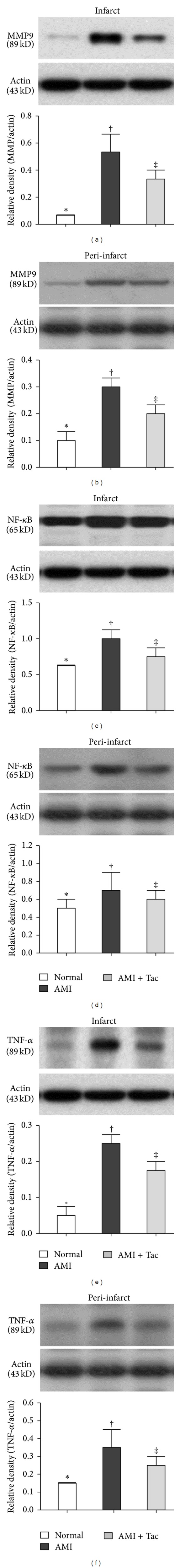
Western blotting for assessing protein expressions of inflammatory biomarkers including MMP-9 (a), (b), NF-*κ*B (c), (d), and TNF-*α* (e), (f) in normal remote myocardium, infarct and peri-infarct areas of both groups. Note the significantly lower expressions of MMP-9 and TNF-*α* in normal than in controls and tac-treated animals (∗ versus other groups, all *P* < 0.001), and significantly higher expressions of these two biomarkers in controls than in tac-treated animals († versus ‡, all *P* < 0.01) (a), (b), (e), (f). Note the significantly lower expressions of NF-*κ*B in normal than in the infarct areas in both groups and the peri-infarct area in controls (∗ versus †. all *P* < 0.05). NF-*κ*B expression is significantly lower in the peri-infarct areas in tac-treated animals (∗ versus †. *P* < 0.05) but there is no significant differences between two groups in the infarct areas (c), (d).

**Figure 7:**
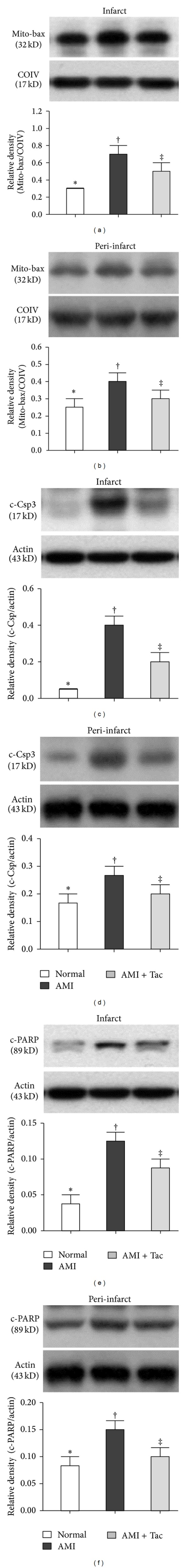
Western blotting for assessing protein expressions of apoptotic biomarkers including Bax (a), (b) and Csp-3 (c), (d), and DNA damage biomarker c-PARP (e), (f) in normal remote myocardium, infarct, and peri-infarct areas of both groups. Note the significantly lower expressions of Bax, Csp-3, and c-PARP in normal than in controls and tac-treated animals (∗ versus other groups, all *P* < 0.05), and significantly higher expressions of these biomarkers in controls than in tac-treated animals († versus ‡, all *P* < 0.05) (a)–(f).

Compared with remote normal myocardium, the protein expression levels of inflammatory biomarkers (MMP-9, NF-*κ*B, and TNF-*α*) (Figure 6), apoptotic biomarkers (Bax and Csp-3), and DNA damage biomarkers (c-PARP) (Figure 7) in the infarct and peri-infarct areas were significantly higher in the control group and tac-treated group. However, there were significantly lower protein expressions of inflammatory (MMP-9, NF-*κ*B, and TNF-*α*), apoptotic, and DNA damage (Bax, Csp-3, c-PARP) biomarkers in the infarct and/or peri-infarct areas in the tac-treated group than in the control group.

## 4. Discussion

CMR is feasible for assessing LVF in experimental and clinical studies [12–14]. The present study supports that intracoronary tacrolimus therapy harboring therapeutic potential against AMI in minipigs by monitoring* in vivo* temporal changes of infarct on serial CMR examinations [9–11]. Our results revealed significant progressive impairment of LVF and contractility on CMR from day 2 to day 21 among controls. In contrast, the tac-treated animals exhibited no significant changes in LVF, contractility, and infarct transmurality between day 2 and day 5, which would strongly suggest that even without reperfusion, early intracoronary tacrolimus therapy can ameliorate AMI damage. Furthermore, even though only a single dose was administered, the ongoing therapeutic effects of tacrolimus extended to day 21 with preservation of LVF and contractility, and alleviation of infarct transmurality and extent.

LGE study of CMR is an accurate technique for the detection of MI and measurement of the infarct extent [11–16]. In the present study, LGE revealed a significant reduction in the infarct extent following intracoronary administration of tacrolimus. These findings showed good correlation with those from postmortem evaluation using TTC staining and histological assessment of myocardial fibrosis. Tacrolimus immunosuppression with resultant reduction of systemic endothelin-1, promotion of microvascular endothelial function, and increment in the mean vessel area over time with positive vascular remodeling have been reported in heart transplant recipients [8]. Our results showed that all minipigs in the control group exhibited D5/D2 IR > 1 in LGE study with further elevated D21/D2 IR, indicating progression of myocardial ischemic injury and infarct size. Diminution of D5/D2 and D21/D2 IR after tacrolimus treatment may reflect better myocardial salvage. The finding is consistent with that of IHC study showing better preservation of small vessels in the infarct and peri-infarct areas in tac-treated animals compared to that in the untreated controls. We believe that D5/D2 IR < 1 might be regarded as an indicator of early regressive changes in AMI after tacrolimus therapy.

Tacrolimus has been proposed as the most effective immunosuppressive drug for the treatment of ischemic stroke. Studies have shown that tacrolimus can reduce the extent of infarct through a neuroprotective effect dependent on mitochondrial protection and downregulation of proinflammatory cytokines [5, 17]. Prior studies have also demonstrated that inflammatory cells accumulating in the MI areas can deliver cytokines and specific antibodies leading to further myocardial damage. Subsequent inflammatory and immune responses can trigger the complement cascade and enhance ROS generation participating in the pathogenesis of adverse ventricular remodeling [4, 9]. On the other hand, the macrophage has also been described as a primary responder cell type involved in the regulation of post-MI wound healing at multiple levels [18]. In the present study, IHC and IHF staining revealed significantly more CD68+ cells (macrophages) and CD40+ cells (inflammatory) in the infarct and peri-infarct regions than in remote normal myocardium. However, intracoronary tacrolimus therapy allowed significant reduction of the macrophages and inflammatory cells. Moreover, the tac-treated animals exhibited a significantly lower level of protein expression of inflammatory biomarkers (MMP-9, NF-*κ*B, and TNF-*α*) in the infarct and/or peri-infarct areas, further verifying the anti-inflammatory effect of intracoronary tacrolimus treatment against AMI [9, 10].

Apoptosis is recognized as a mechanism of cell loss in AMI [19]. The release of mitochondrial cytochrome C accentuates apoptotic cell death through caspase activation in brain ischemia. Accordingly, tacrolimus has been shown to be effective in inhibiting cytochrome C release and Csp-3 activation, thus leading to mitochondria protection [20]. Our results demonstrated that the tac-treated minipigs exhibited significantly lower protein expressions of Bax and Csp-3 and c-PARP than those in controls. This would seem to support the claim that intracoronary tacrolimus therapy could contribute to alleviation of AMI-related cellular apoptosis and DNA damages, thereby allowing preservation of LV mass [21, 22].

Our present study also has limitations. First, a limited size sample of 6 minipigs was used in each group. Second, identical positioning in serial assessments may be difficult for the clinical and physiologic status of minipigs could not be exactly the same during the experiment period. Third, the differences in histopathology, immunostaining, and western blotting between 2 groups on day 2 and day 5 were not known. Fourth, although the effect of the tacrolimus on AMI was substantial based on the presented data and several articles reporting that intracoronary tacrolimus could alleviate AMI by attenuating oxidative stress, inflammatory responses, and apoptosis, regulate mitogen-activeted protein kinase and Akt signaling pathway, and suppress the inappropriate innate immunity, the exact underlying mechanisms involved have not been fully clarified [9–11]. On the other hand, recent studies reported that everolimus treatment induced inhibition of translation in both cultured macrophages and smooth muscle cells. However, cell death occurred only in macrophages and was characterized by bulk degradation of long-lived proteins, processing of microtubule-associated protein light chain 3, and cytoplasmic vacuolization, which are all markers of autophagy. Everolimus induced autophagy by mediating mTOR inhibition while cell viability was not affected using tacrolimus, an mTOR-independent everolimus analog [23, 24]. Finally, this swine model is not identical to the clinical scenario in which most patients with AMI have a reperfusion of the culprit artery. However, in order to assess the role of tacrolimus against AMI, a nonreperfused model was deliberately designed in the present study to eliminate the effect of reperfusion. Further studies are needed to evaluate the role of tacrolimus and CMR changes in a reperfused model but a meticulous design is needed to differentiate whether the improvement of LV function is related to tacrolimus or reperfusion per se.

In conclusion, CMR demonstrated preservation of LVF and contractility and significantly smaller infarct* in vivo* after AMI following tacrolimus treatment in a swine model. The results correlated with* in vitro* findings at histological and protein levels.

## Figures and Tables

**Table 1:** Cardiac magnetic resonance parameters of CINE and late gadolinium enhancement for assessing minipigs with acute myocardial infarction (AMI) without and with intracoronary tacrolimus treatment.

	CINE	LGE
Inversion time (msec)	NA	200–280
Flip angle	55°	15°
Echo time (msec)	Minfull	Minimum
Bandwidth (kHz)	125	31.25
RR-interval	NA	2
Trigger delay (msec)	NA	255
Thickness/gap (mm)	8/2	8/2
Field of view (mm)	280 × 280	280 × 280
Frequency matrix	224	160
Phase matrix	192	160
Number of excitation	1	1
Phase field of view	0.8	1
Views per second	16	8
Number of cardiac phase	20	1

NA: not applicable.

**Table 2:** Comparisons of  left ventricular (LV) function and late gadolinium enhancement (LGE) in AMI minipigs without and with tacrolimus therapy.

	Control Mean ± SD	Tac-treated Mean ± SD	*P*-value^∗^
LV mass/BW ratio (g/kg)			
Day 2	2.65 ± 0.05	2.64 ± 0.06	0.907
Day 5	2.62 ± 0.05	2.62 ± 0.10	0.848
Day 21	2.30 ± 0.06	2.56 ± 0.05	0.002
*P*_1_ value (D2 versus D5)	*0.188 *	*0.438 *	
*P*_2_ value (D2 versus D21)	*0.031 *	*0.094 *	
*P*_3_ value (D5 versus D21)	*0.031 *	*0.313 *	
ED volume/BW ratio (mL/kg)			
Day 2	3.07 ± 0.16	3.08 ± 0.14	0.976
Day 5	3.23 ± 0.10	3.19 ± 0.12	0.167
Day 21	3.46 ± 0.14	3.01 ± 0.11	0.002
*P*_1_ value (D2 versus D5)	*0.031 *	*0.156 *	
*P*_2_ value (D2 versus D21)	*0.031 *	*0.531 *	
*P*_3_ value (D5 versus D21)	*0.188 *	*0.031 *	
ES volume/BW ratio (mL/kg)			
Day 2	1.47 ± 0.09	1.39 ± 0.10	0.394
Day 5	1.77 ± 0.10	1.54 ± 0.09	0.006
Day 21	2.13 ± 0.18	1.38 ± 0.14	0.002
*P*_1_ value (D2 versus D5)	*0.031 *	*0.063 *	
*P*_2_ value (D2 versus D21)	*0.031 *	*0.844 *	
*P*_3_ value (D5 versus D21)	*0.031 *	*0.031 *	
Stroke volume (mL)			
Day 2	26.72 ± 1.68	27.78 ± 1.20	0.331
Day 5	26.08 ± 1.82	27.78 ± 0.69	0.180
Day 21	22.87 ± 1.86	29.98 ± 2.77	0.002
*P*_1_ value (D2 versus D5)	*0.844 *	*0.563 *	
*P*_2_ value (D2 versus D21)	*0.031 *	*0.219 *	
*P*_3_ value (D5 versus D21)	*0.031 *	*0.094 *	
Ejection fraction (%)			
Day 2	53.00 ± 2.68	54.78 ± 2.27	0.258
Day 5	47.13 ± 2.33	52.55 ± 1.75	0.004
Day 21	38.57 ± 3.46	54.82 ± 4.69	0.002
*P*_1_ value (D2 versus D5)	*0.031 *	*0.156 *	
*P*_2_ value (D2 versus D21)	*0.031 *	*0.844 *	
*P*_3_ value (D5 versus D21)	*0.031 *	*0.219 *	
Infarct/LV area on LGE (%)			
Day 2	14.93 ± 1.23	14.84 ± 1.15	0.967
Day 5	15.85 ± 0.85	14.15 ± 0.87	0.013
Day 21	16.75 ± 0.67	12.05 ± 0.82	0.001
*P*_1_ value (D2 versus D5)	*0.031 *	*0.173 *	
*P*_2_ value (D2 versus D21)	*0.031 *	*0.031 *	
*P*_3_ value (D5 versus D21)	*0.031 *	*0.031 *	

BW: body weight; D: post operation day; ED: end diastolic; ES: end systolic; *P*_1_, *P*_2_, *P*_3_ values: comparisons between D2 versus D5, D2 versus D21, and D5 versus D21, respectively.

*P* value^∗^ and *P*_1–3_ values were exact *P* values using Mann-Whitney test∗ and Wilcoxon signed-rank test, respectively.

**Table 3:** Comparisons of degree of fibrosis, vascular densities, and inflammatory cells in explanted heart of minipigs without and with tacrolimus therapy.

	Normal Mean ± SD	Infarct		Peri-infarct		*P* value∗
		Control Mean ± SD	Tac-treated Mean ± SD	Control Mean ± SD	Tac-treated Mean ± SD	
Mean fibrotic areas (×10^4^ *μ*m^2^/HPF)	1.2 ± 0.1	384.1 ± 8.9	276.3 ± 23.6	31.5 ± 9.5	13.1 ± 0.5	*P* < 0.001
		*P*_1_ * = 0.013 *		*P*_2_ * = 0.002 *		

Number of small vessels/HPF	8.1 ± 0.4	2.5 ± 0.4	4.2 ± 0.4	4.8 ± 0.1	6.6 ± 0.5	*P* < 0.05
		*P*_1_ * = 0.031 *		*P*_2_ * = 0.023 *		

% of CD68+ cells	0.3 ± 0.0	8.0 ± 0.2	6.3 ± 0.2	3.7 ± 0.4	2.2 ± 0.5	*P* < 0.001
		*P*_1_ * = 0.019 *		*P*_2_ * = 0.027 *		

% of CD40+ cells	5.8 ± 1.2	68.8 ± 3.8	51.2 ± 2.9	47.1 ± 2.0	36.7 ± 2.4	*P* < 0.001
		*P*_1_ * = 0.021 *		*P*_2_ * = 0.031 *		

HFP: high power field; *P* value^∗^: normal remote myocardium versus other groups; *P*_1_, *P*_2_: comparisons in infarct and peri-infarct areas, respectively.

(Statistical analyses using one-way ANOVA followed by Tukey's multiple comparison procedure with significance at 0.05 level).
